# Supplementary material for: Covalent PARylation of DNA base excision repair proteins regulates DNA demethylation
Source: Nat Commun. 2024 Jan 2;15:184. doi: 10.1038/s41467-023-44209-8 (PMC10762122; doi:10.1038/s41467-023-44209-8)
Supplement: Supplementary file 1 — Supplementary Information [file 41467_2023_44209_MOESM1_ESM.pdf]

Supplementary Data for

Covalent PARylation of DNA base excision repair proteins regulates DNA demethylation

Authors:

Simon D. Schwarz[1*], Jianming Xu [1,2]* Kapila Gunasekera[2,3], David Schürmann[1], Cathrine B. Vågbø[4], Elena Ferrari[2], Geir Slupphaug [4], Michael O. Hottiger[2], Primo Schär[1,#], Roland Steinacher[1, 5,#]

Affiliations:

[1]Department of Biomedicine, University of Basel; Mattenstrasse 28, 4058 Basel, Switzerland.

[2]Department of Molecular Mechanisms of Disease; University of Zurich, Winterthurerstrasse 190, 8057 Zurich, Switzerland.

[3]Department of Chemistry, Biochemistry and Pharmaceutical Sciences, University of Bern, Freiestrasse 3, 3012 Bern, Switzerland.

[4] Proteomics and Modomics Experimental Core Facility (PROMEC), Norwegian University of Science and Technology and St. Olavs Hospital, 7491 Trondheim, Norway

[5] Institute of Molecular Health Sciences, ETH Zurich, Otto-Stern-Weg 7
8093 Zurich, Switzerland.

*These authors contributed equally to this work

[#] Corresponding authors: Primo.Schaer@unibas.ch, Roland.Steinacher@biol.ethz.ch

This PDF file includes:

Supplementary figures 1 to 4

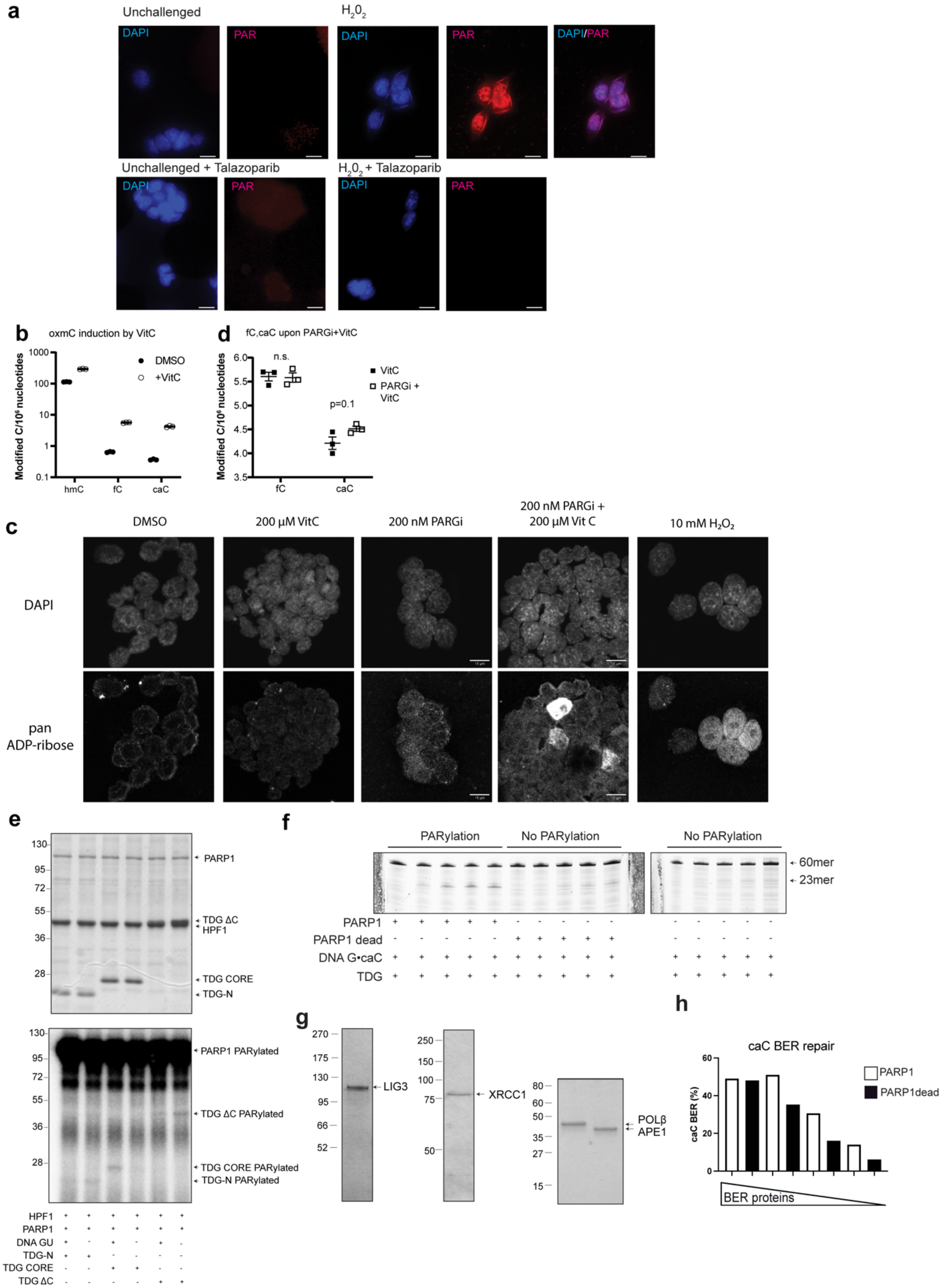
a
Unchallenged
H20 2
DAPI
PAR
DAPI/PAR
Unchallenged + Talazoparib
H20 2 + Talazoparib
b
oxmC induction by VitC
Modified C/10⁶ nucleotides
DMSO
+VitC
hmC
fC
caC
d
fC,caC upon PARGi+VitC
n.s.
p=0.1
VitC
PARGi + VitC
c
DMSO
200 µM VitC
200 nM PARGi
200 nM PARGi + 200 µM Vit C
10 mM H2O2
pan ADP-ribose
e
PARP1
TDG ΔC
HPF1
TDG CORE
TDG-N
PARP1 PARylated
TDG ΔC PARylated
TDG CORE PARylated
TDG-N PARylated
HPF1
PARP1
DNA GU
TDG-N
TDG CORE
TDG ΔC
f
PARylation
No PARylation
60mer
23mer
PARP1
PARP1 dead
DNA G•caC
TDG
g
LIG3
XRCC1
POLβ
APE1
h
caC BER repair
caC BER (%)
PARP1
PARP1dead
BER proteins

Supplementary Fig. 1
Covalent PARylation of BER proteins stimulates DNA demethylation-associated caC-excision
a, Immunofluorescence staining showing DAPI and PAR signals in mESC either unchallenged or treated with H_2O_2 (1mM) with and without Talazoparib (100 nM) treatment. Scale bar = 10 µm **b,** Mass spectrometry measurements of oxmCs upon 200 µM VitC (16 h) (mean +/- SEM, n=3 independent experiments) **c,** detection of pan-ADP-ribosylation via immunofluorescence using the detection reagent MABE1016. Displayed images are summed intensity z-projections of confocal stacks. Images were acquired using the same settings and all images were set to the same dynamic range of grayscales. **d,** Mass spectrometry measurements of fC and caC in TDGwt ESCs treated with 200 µM VitC and 25 nM PARGi for 16 h. (mean +/- SEM, n=3) **e,** In vitro PARylation of TDG. Purified domains of human TDG domains (0.4 µM) were incubated with PARP1 (0.4 µM) and HPF1 (2 µM) in the presence of [32P]-NAD, homoduplex DNA oligomer (200 nM) and G•U-containing DNA oligonucleotides (400 nM) as indicated. Samples were separated by SDS-PAGE and analyzed by Coomassie blue (upper panel) and autoradiography (lower panel). **f,** TDG caC excision reaction **g,** Coomassie-stained SDS-PAGE showing recombinant purified BER proteins as indicated. **h,** Quantitation of reconstituted caC excision and repair reaction with recombinant TDG, APE1, POLβ, XRCC1 and LIG3 and PARP1 or catalytic dead PARP1 (PARP1dead). Source data are provided in the Source Data file.

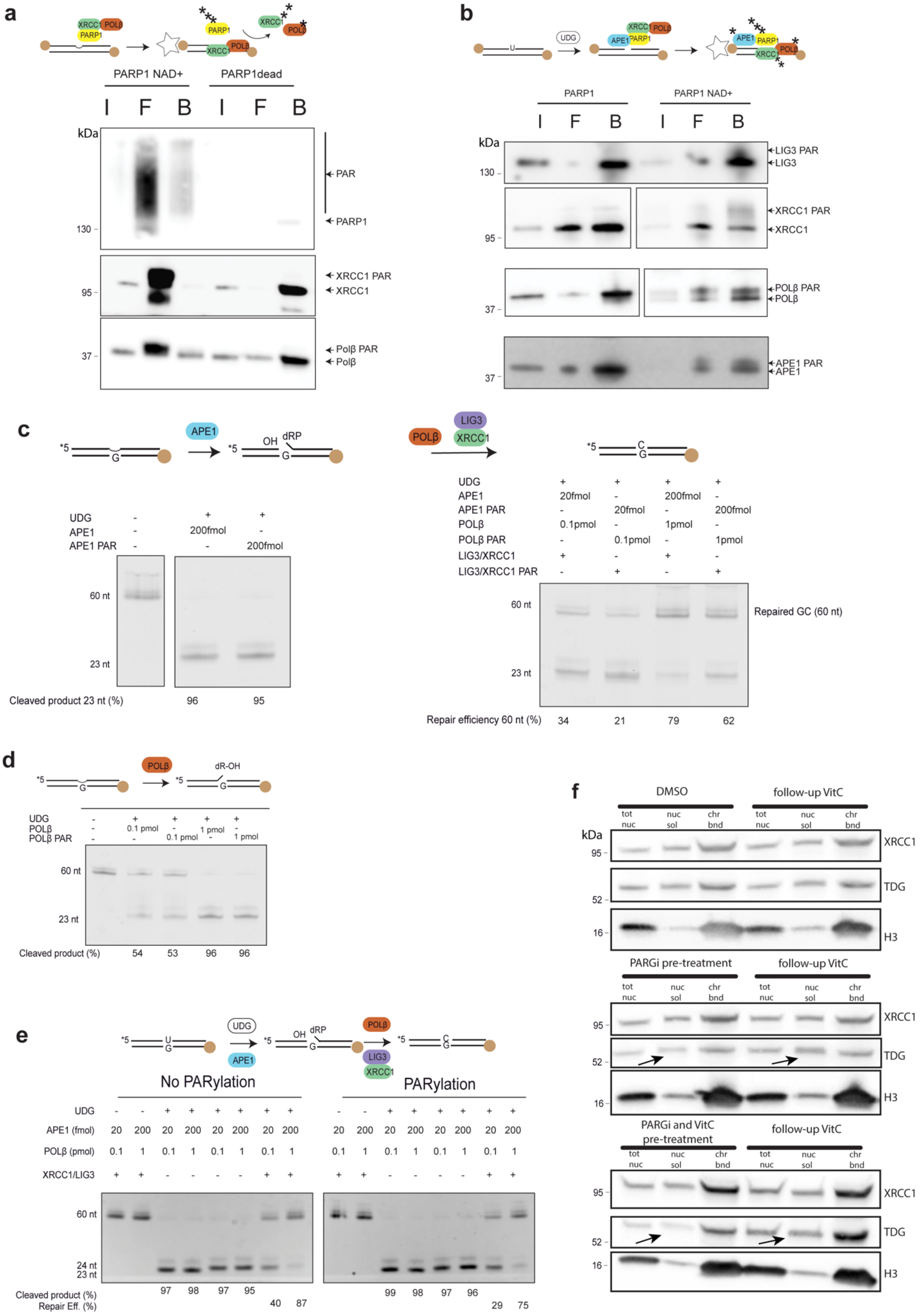

a
XRCC1
POLβ
PARP1
PARP1 NAD+
PARP1dead
I F B I F B
kDa
130
95
37
PAR
PARP1
XRCC1 PAR
XRCC1
Polβ PAR
Polβ
b
UDG
APE1
LIG3
PARP1
PARP1 NAD+
LIG3 PAR
LIG3
XRCC1 PAR
POLβ PAR
POLβ
APE1 PAR
c
*5
G
OH
dRP
C
UDG
APE1
APE1 PAR
200fmol
60 nt
23 nt
Cleaved product 23 nt (%)
96
95
20fmol
POLβ
POLβ PAR
0.1pmol
1pmol
LIG3/XRCC1
LIG3/XRCC1 PAR
Repaired GC (60 nt)
Repair efficiency 60 nt (%)
34
21
79
62
d
dR-OH
0.1 pmol
1 pmol
Cleaved product (%)
54
53
96
96
e
U
No PARylation
PARylation
APE1 (fmol)
POLβ (pmol)
XRCC1/LIG3
24 nt
Cleaved product (%)
Repair Eff. (%)
97
98
97
95
40
87
99
98
97
96
29
75
f
DMSO
follow-up VitC
tot nuc
nuc sol
chr bnd
kDa
52
16
XRCC1
TDG
H3
PARGi pre-treatment
PARGi and VitC pre-treatment

Supplementary Fig. 2

AP-site and SSB activate PARP1 to PARylate BER proteins that then dissociate from DNA
a, XRCC1-POLβ AP-site binding experiments and immunoblot analysis. Purified XRCC1 and POLβ proteins (0.5 μM) bound to AP-site containing DNA (1 μM) immobilized on streptavidin beads (star), incubated with PARP1 (0.5 μM) and HPF1 (0.5 μM) as indicated. I, input; F, flow; B, DNA bound. **b,** BER protein SSB interaction experiment and immunoblot analysis. Purified XRCC1, LIG3, POLβ and APE1 proteins (0.5 μM) bound to AP-site containing DNA substrate (1 μM) immobilized on streptavidin beads (star) were incubated with PARP1 (0.5 μM) and HPF1 (0.5 μM) and the immobilized protein-DNA complex washed with low salt buffer. I, input; F, flow; B, bound. **c,** AP-site incision (left panel) and repair assays (right panel). A 60mer G•U-containing substrate (100 nM) was digested with UDG to generate an AP-site. AP-site cleavage efficiency (cleaved product %) is shown (left). AP-site repair by APE1, APE1 PARylated, POLβ, POLβ PARylated, XRCC1, XRCC1 PARylated, LIG3, LIG3 PARylated is shown as repair efficiency (%). **d,** POLβ lyase assay. AP-site cleavage efficiency (cleaved product %) by POLβ, POLβ PARylated is shown. **e,** G•U BER repair assay. Reconstitution of the G•U BER reaction with UDG, APE1, POLβ and PARylated POLβ, XRCC1 and LIG3. AP-site cleavage efficiency (cleaved product %) and repair efficiency (Repair eff. %) is shown. **f,** Nuclear fractionations of mESC in conditions indicated. tot nuc: total nuclear fraction, nuc sol: nuclear soluble fraction, chr bnd: chromatin bound fraction. Arrows point to most visible change. Source data are provided in the Source Data file.

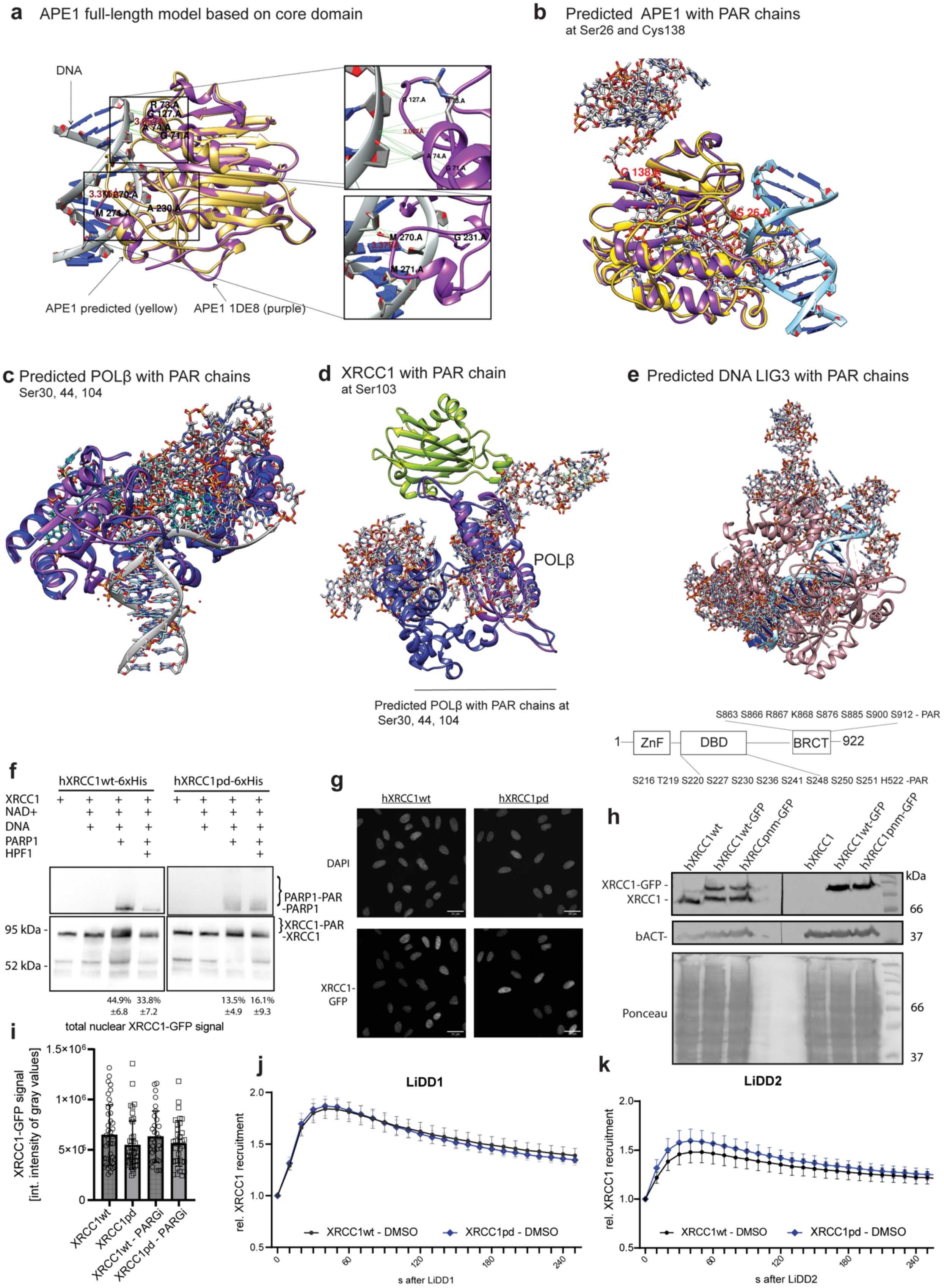
a APE1 full-length model based on core domain
DNA
APE1 predicted (yellow)
APE1 1DE8 (purple)
b Predicted APE1 with PAR chains
at Ser26 and Cys138
C 138.A
S 26.A
c Predicted POLβ with PAR chains
Ser30, 44, 104
d XRCC1 with PAR chain
at Ser103
POLβ
Predicted POLβ with PAR chains at
Ser30, 44, 104
e Predicted DNA LIG3 with PAR chains
S863 S866 R867 K868 S876 S885 S900 S912 - PAR
1 ZnF DBD BRCT 922
S216 T219 S220 S227 S230 S236 S241 S248 S250 S251 H522 -PAR
f
hXRCC1wt-6xHis
hXRCC1pd-6xHis
XRCC1
NAD+
DNA
PARP1
HPF1
PARP1-PAR
-PARP1
XRCC1-PAR
-XRCC1
95 kDa -
52 kDa -
44.9% ±6.8
33.8% ±7.2
13.5% ±4.9
16.1% ±9.3
g
hXRCC1wt
hXRCC1pd
DAPI
XRCC1-GFP
h
hXRCC1wt
hXRCC1wt-GFP
hXRCCpnm-GFP
hXRCC1
hXRCC1wt-GFP
hXRCC1pnm-GFP
kDa
XRCC1-GFP -
XRCC1 -
66
bACT-
37
Ponceau
66
37
i
total nuclear XRCC1-GFP signal
XRCC1-GFP signal [int. intensity of gray values]
1.5×10⁶
1×10⁶
5×10⁵
0
XRCC1wt
XRCC1pd
XRCC1wt - PARGi
XRCC1pd - PARGi
j
LiDD1
rel. XRCC1 recruitment
s after LiDD1
XRCC1wt - DMSO
XRCC1pd - DMSO
k
LiDD2
rel. XRCC1 recruitment
s after LiDD2
XRCC1wt - DMSO
XRCC1pd - DMSO

Supplementary Fig. 3
Modelled covalent M/PARylation interferes with DNA binding and PARylation of hXRCC1 reduces recruitment to damage in U2OS cells
a, and b, Protein structures of ADP-ribosylated human APE1, showing the overlap (>95%) of the modelled full-length (PDB: 1DE8) and the published core (PDB: 8ICZ) protein structures. Magnifications show amino acid residues involved in DNA contacts. Hydrogen bonds are shown in green. PAR chains were modelled to the indicated amino acids **c,** Protein structure of POLβ with PAR chains modelled to indicated Serines. **d,** Modelled PARylation to the co-structure of POLβ bound to XRCC1 (PDB: 3LQC). **e,** Structure of human DNA ligase 3 (LIG3, PDB: 3L2P) with zinc finger domain (ZnF) and the DNA binding domain (DBD) with modelled PARylation as indicated. **f,** Representative image of in-vitro PARylated, recombinant hXRCC1wt and hXRCC1pd under indicated conditions. Signal stems from probing with anti-XRCC1 antibody (Sigma, X0629). A densitometric measurement of PARylated vs. non-PARylated XRCC1, per lane, is indicated below as mean +/- SEM of n=3 reactions with hXRCC1 variants from 3 purifications each. **g,** Representative images of polyclonal U2OS cell population harbouring the indicated hXRCC-GFP constructs. **h,** Immunodetection of hXRCC (left) and GFP (Roche 180303, right). Ectopic hXRCC1 expression reaches 70% of the endogenous hXRCC1 in both cases. **i,** Integrated GFP intensity of all ESC per condition investigated in LiDD experiments (mean+/-SD). **j,** Mean recruitment to site of first damage (LiDD1) in U2OS cells treated with DMSO **k,** Mean re-recruitment to site of second damage (LiDD2). Error bars indicate 95% confidence-interval based on n=40(wt) and n=44(pd) cells from 5 independent experiments. Source data are provided in the Source Data file.

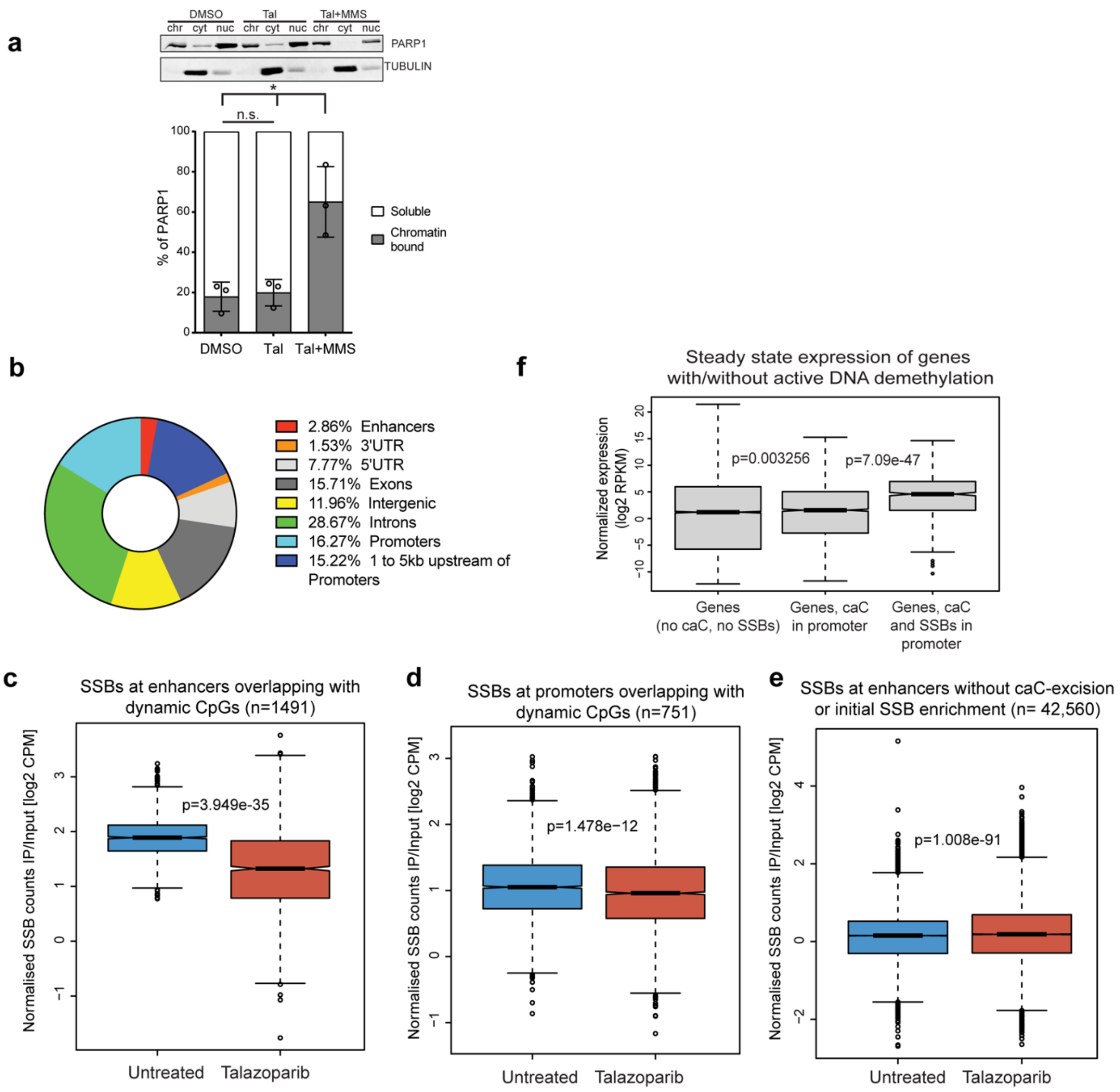
a
DMSO
Tal
Tal+MMS
chr cyt nuc chr cyt nuc chr cyt nuc
PARP1
TUBULIN
*
n.s.
% of PARP1
Soluble
Chromatin bound
DMSO
Tal
Tal+MMS
b
2.86% Enhancers
1.53% 3'UTR
7.77% 5'UTR
15.71% Exons
11.96% Intergenic
28.67% Introns
16.27% Promoters
15.22% 1 to 5kb upstream of Promoters
f
Steady state expression of genes with/without active DNA demethylation
Normalized expression (log2 RPKM)
p=0.003256
p=7.09e-47
Genes (no caC, no SSBs)
Genes, caC in promoter
Genes, caC and SSBs in promoter
c
SSBs at enhancers overlapping with dynamic CpGs (n=1491)
Normalised SSB counts IP/Input [log2 CPM]
p=3.949e-35
Untreated
Talazoparib
d
SSBs at promoters overlapping with dynamic CpGs (n=751)
Normalised SSB counts IP/Input [log2 CPM]
p=1.478e−12
Untreated
Talazoparib
e
SSBs at enhancers without caC-excision or initial SSB enrichment (n= 42,560)
Normalised SSB counts IP/Input [log2 CPM]
p=1.008e-91
Untreated
Talazoparib

Supplementary Fig. 4
PARylation promotes targeted BER in DNA demethylation and the repair of random SSBs in mESC
a, Chromatin association of PARP1 upon Talazoparib Top: Representative immunoblot image of fractionated ESCs upon 5 nM Tal (and 0.01% MMS) probed for PARP1 and Tubulin. Chr: chromatin bound, cyt: cytoplasmic, nuc: nuclear soluble. Bottom: Quantification of chromatin bound or soluble PARP1. Depicted is the mean +/- SD of n=3. *: $P<0.05$, two-tailed t-test **b,** Relative representation (%) of SSB-enriched regions in genomic locations of wildtype mESC treated with DMSO. **c,** Normalized SSB read counts per conditions at enhancers displaying dynamic CpGs and SSB-enrichment in unchallenged ESCs. **d,** Normalized SSB read counts in promoters displaying dynamic CpGs and SSB-enrichment in unchallenged mESC. **e,** Normalized SSB read counts in enhancers without either dynamic CpGs or initial SSB enrichment un unchallenged mESC. **f,** Normalized expression (log2 RPKM) of genes without TDG caC excision or SSB-enrichment in the promoter of genes, only with caC excision in their promoters and of genes with caC excision and SSBs in their promoter. **c-f** p-values from two-sided Wilcoxon test, Source data are provided in the Source Data file.
