# Supplementary material for: Covalent PARylation of DNA base excision repair proteins regulates DNA demethylation
Source: Nat Commun. 2024 Jan 2;15:184. doi: 10.1038/s41467-023-44209-8 (PMC10762122; doi:10.1038/s41467-023-44209-8)
Supplement: Supplementary file 3 — Reporting Summary [file 41467_2023_44209_MOESM3_ESM.pdf]

Corresponding author(s): Roland Steinacher, Primo Schär

Last updated by author(s): Nov 14, 2023

Reporting Summary

Nature Portfolio wishes to improve the reproducibility of the work that we publish. This form provides structure for consistency and transparency in reporting. For further information on Nature Portfolio policies, see our Editorial Policies and the Editorial Policy Checklist.

Statistics

For all statistical analyses, confirm that the following items are present in the figure legend, table legend, main text, or Methods section.

n/a	Confirmed	
☐	☒	The exact sample size (*n*) for each experimental group/condition, given as a discrete number and unit of measurement
☐	☒	A statement on whether measurements were taken from distinct samples or whether the same sample was measured repeatedly
☐	☒	The statistical test(s) used AND whether they are one- or two-sided<br>*Only common tests should be described solely by name; describe more complex techniques in the Methods section.*
☒	☐	A description of all covariates tested
☒	☐	A description of any assumptions or corrections, such as tests of normality and adjustment for multiple comparisons
☐	☒	A full description of the statistical parameters including central tendency (e.g. means) or other basic estimates (e.g. regression coefficient) AND variation (e.g. standard deviation) or associated estimates of uncertainty (e.g. confidence intervals)
☐	☒	For null hypothesis testing, the test statistic (e.g. *F*, *t*, *r*) with confidence intervals, effect sizes, degrees of freedom and *P* value noted<br>*Give P values as exact values whenever suitable.*
☒	☐	For Bayesian analysis, information on the choice of priors and Markov chain Monte Carlo settings
☒	☐	For hierarchical and complex designs, identification of the appropriate level for tests and full reporting of outcomes
☒	☐	Estimates of effect sizes (e.g. Cohen's *d*, Pearson's *r*), indicating how they were calculated

Our web collection on statistics for biologists contains articles on many of the points above.

Software and code

Policy information about availability of computer code

Data collection	Chemiluminescence detection with PXi 6 imaging system GeneSys (V1.8.2.0), Syngene.<br>Fluorescence acquisition (Gels) with blue fluorescence mode of the Typhoon 9400 (GE Healthcare).<br>Fluorescence acquisition in cells with Leica DMI 4000 and LAS X 3.3.3.16958<br>Sequencing was performed on a Illumina NextSeq platform<br>Agilent 1290 Infinity II UHPLC system with an ZORBAX RRHD Eclipse Plus C18 150 x 2.1 mm (1.8 µm) column protected with an ZORBAX RRHD Eclipse Plus C18 5 x 2.1 mm (1.8 µm) guard column (Agilent) was used for chromatographic separation.<br>Mass spectrometric detection was performed using an Agilent 6495 Triple Quadrupole system operating in positive electrospray ionization mode.<br>confocal microscopy system consists of a fully automated Nikon Ti-E microscope, equipped with a Hamamtsu Flash 4.0 V2 CMOS camera and a passively Q-switched 355 nm UV-A ablation laser (2D-VisiFRAP-DC, Visitron Systems GmbH).
Data analysis	Quantification of signal intensity with Fiji ImageJ 2.0.0-rc-69/1.53c<br>Bioinformatical analyses were performed with the following softwares and packages:<br>bowtie2 V2.3.4.2, picard tools V2.9.2, HOMER V4.11, STAR Aligner V2.6, HTseq V0.12.4<br>R V3.6 with packages and dependencies: Rsamtools V 2.10, bamsignals V1.22.0, edgeR V3.13, ChIPseeker V 1.26, DESeq2 version 1.26.0<br>GraphPad Prism V 9.5.1

For manuscripts utilizing custom algorithms or software that are central to the research but not yet described in published literature, software must be made available to editors and reviewers. We strongly encourage code deposition in a community repository (e.g. GitHub). See the Nature Portfolio guidelines for submitting code & software for further information.

Data

Policy information about availability of data

All manuscripts must include a data availability statement. This statement should provide the following information, where applicable:

- Accession codes, unique identifiers, or web links for publicly available datasets
- A description of any restrictions on data availability
- For clinical datasets or third party data, please ensure that the statement adheres to our policy

Source Data is provided for this work.
The SSB-seq and RNA-Seq data can be found on the gene expression omnibus (GEO) under the accession number GSE166963 (https://www.ncbi.nlm.nih.gov/geo/query/acc.cgi?acc=GSE166963) or the BioProject PRJNA743896 (SRR15047557-559, https://www.ncbi.nlm.nih.gov/bioproject/?term=PRJNA743896), respectively.

Research involving human participants, their data, or biological material

Policy information about studies with human participants or human data. See also policy information about sex, gender (identity/presentation), and sexual orientation and race, ethnicity and racism.

Reporting on sex and gender	n/a
Reporting on race, ethnicity, or other socially relevant groupings	n/a
Population characteristics	n/a
Recruitment	n/a
Ethics oversight	n/a

Note that full information on the approval of the study protocol must also be provided in the manuscript.

Field-specific reporting

Please select the one below that is the best fit for your research. If you are not sure, read the appropriate sections before making your selection.

☒ Life sciences ☐ Behavioural & social sciences ☐ Ecological, evolutionary & environmental sciences

For a reference copy of the document with all sections, see nature.com/documents/nr-reporting-summary-flat.pdf

Life sciences study design

All studies must disclose on these points even when the disclosure is negative.

Sample size	No statistical calculations were performed to assess sample sizes. Three independent biological replicates were used in general, as commonly accepted in the field to reduce the chance of outlier measurements and work economically. Differing sample sizes are indicated in alternative comparisons.
Data exclusions	No data was excluded
Replication	Experiments were usually repeated (at least) three times to ensure reproducibility. For measurements with very high variation (e.g. cell-to-cell variation, more replicates were assessed.
Randomization	No randomi
Blinding	No blinding was applied as data were measured with the same objective/digital tools, over all samples and conditions. No counting by hand/subjective estimation was used that could bias results.

Reporting for specific materials, systems and methods

We require information from authors about some types of materials, experimental systems and methods used in many studies. Here, indicate whether each material, system or method listed is relevant to your study. If you are not sure if a list item applies to your research, read the appropriate section before selecting a response.

Materials & experimental systems

n/a	Involved in the study
☐	☒ Antibodies
☐	☒ Eukaryotic cell lines
☐	☐ Palaeontology and archaeology
☐	☐ Animals and other organisms
☐	☐ Clinical data
☐	☐ Dual use research of concern
☐	☐ Plants

Methods

n/a	Involved in the study
☐	☒ ChIP-seq
☐	☐ Flow cytometry
☐	☐ MRI-based neuroimaging

Antibodies

Antibodies used

anti-PAR (Clone 10H, Sigma),
Cy3-conjugated goat anti-mouse secondary Ab (Jackson ImmunoResearch)
anti-PARP1 (Abcam, ab32138)
anti-XRCC1 (Sigma-Aldrich, X0629)
anti-POLβ (Acris, AM00275PU-N)
anti-PAR (Trevigen 4336-BPC-100)
anti-LIG3 (Genetex (6G9) GTX70147)
anti-APEX1 (Invitrogen, MA1-440 (13B 8E5C2)),
anti-DIG (Roche,11333089001)
anti-MAR/PAR (Sigma-Aldrich, MABE1016)
anti-histone H3 (Abcam, 1791)
anti-betaActin (Sigma-Aldrich, A5441)
ECL anti-rabbit IgG HRP-linked (donkey, VWR NA934-1ML)
ECL anti-mouse IgG HRP-linked (sheep, VWR NXA931-1ML)
used dilutions can be found in the manuscript

Validation

The TDG 141 antibody has been raised agains full length hTDG, and verfied in the Schär lab against human and mouse and was also described in : Steinacher et al. 2019, EMBOJ 38:e99242
All other antibodies have been validated by the manufacturer for the use against the indicated proteins.

Eukaryotic cell lines

Policy information about cell lines and Sex and Gender in Research

Cell line source(s)

ESCs were derived from male blastocysts from timed matings of male miniTdg(tg/tg)//Tdg-/- C57BL/6JRj mice with 2 super-ovulated female Tdg+/- mice. Further details can be found at Schwarz SD,F1000Research 2020, 9:1112 (https://doi.org/10.12688/f1000research.25637.2)
U2OS: (RRID: CVCL_0042)

Authentication

ESCs were produced/derived in our laboratory. U2OS cells were originally commissioned (and authenticated) from ATCC (HTB-96) and since then modified in our lab. Documented storage and morphological characterization allow cultivation without misidentification.

Mycoplasma contamination

All cells are frequently tested against mycoplasma contamination, using supernatant of long-term cultivated cells and qPCR analysis by EurofinsGenomics.

Commonly misidentified lines
(See ICLAC register)

no commonly misidentified cell lines were used

Palaeontology and Archaeology

Specimen provenance

Provide provenance information for specimens and describe permits that were obtained for the work (including the name of the issuing authority, the date of issue, and any identifying information). Permits should encompass collection and, where applicable, export.

Specimen deposition

Indicate where the specimens have been deposited to permit free access by other researchers.

Dating methods

If new dates are provided, describe how they were obtained (e.g. collection, storage, sample pretreatment and measurement), where they were obtained (i.e. lab name), the calibration program and the protocol for quality assurance OR state that no new dates are provided.

☐ Tick this box to confirm that the raw and calibrated dates are available in the paper or in Supplementary Information.

Ethics oversight

Identify the organization(s) that approved or provided guidance on the study protocol, OR state that no ethical approval or guidance

Ethics oversight | *was required and explain why not.*

Note that full information on the approval of the study protocol must also be provided in the manuscript.

Animals and other research organisms

Policy information about studies involving animals; ARRIVE guidelines recommended for reporting animal research, and Sex and Gender in Research

Laboratory animals	*For laboratory animals, report species, strain and age OR state that the study did not involve laboratory animals.*
Wild animals	*Provide details on animals observed in or captured in the field; report species and age where possible. Describe how animals were caught and transported and what happened to captive animals after the study (if killed, explain why and describe method; if released, say where and when) OR state that the study did not involve wild animals.*
Reporting on sex	*Indicate if findings apply to only one sex; describe whether sex was considered in study design, methods used for assigning sex. Provide data disaggregated for sex where this information has been collected in the source data as appropriate; provide overall numbers in this Reporting Summary. Please state if this information has not been collected. Report sex-based analyses where performed, justify reasons for lack of sex-based analysis.*
Field-collected samples	*For laboratory work with field-collected samples, describe all relevant parameters such as housing, maintenance, temperature, photoperiod and end-of-experiment protocol OR state that the study did not involve samples collected from the field.*
Ethics oversight	*Identify the organization(s) that approved or provided guidance on the study protocol, OR state that no ethical approval or guidance was required and explain why not.*

Note that full information on the approval of the study protocol must also be provided in the manuscript.

Clinical data

Policy information about clinical studies

All manuscripts should comply with the ICMJE guidelines for publication of clinical research and a completed CONSORT checklist must be included with all submissions.

Clinical trial registration	*Provide the trial registration number from ClinicalTrials.gov or an equivalent agency.*
Study protocol	*Note where the full trial protocol can be accessed OR if not available, explain why.*
Data collection	*Describe the settings and locales of data collection, noting the time periods of recruitment and data collection.*
Outcomes	*Describe how you pre-defined primary and secondary outcome measures and how you assessed these measures.*

Dual use research of concern

Policy information about dual use research of concern

Hazards

Could the accidental, deliberate or reckless misuse of agents or technologies generated in the work, or the application of information presented in the manuscript, pose a threat to:

No	Yes	
☒	☐	Public health
☒	☐	National security
☒	☐	Crops and/or livestock
☒	☐	Ecosystems
☒	☐	Any other significant area

Experiments of concern

Does the work involve any of these experiments of concern:

No	Yes	
☒	☐	Demonstrate how to render a vaccine ineffective
☒	☐	Confer resistance to therapeutically useful antibiotics or antiviral agents
☒	☐	Enhance the virulence of a pathogen or render a nonpathogen virulent
☒	☐	Increase transmissibility of a pathogen
☒	☐	Alter the host range of a pathogen
☒	☐	Enable evasion of diagnostic/detection modalities
☒	☐	Enable the weaponization of a biological agent or toxin
☒	☐	Any other potentially harmful combination of experiments and agents

Plants

Seed stocks	*Report on the source of all seed stocks or other plant material used. If applicable, state the seed stock centre and catalogue number. If plant specimens were collected from the field, describe the collection location, date and sampling procedures.*
Novel plant genotypes	*Describe the methods by which all novel plant genotypes were produced. This includes those generated by transgenic approaches, gene editing, chemical/radiation-based mutagenesis and hybridization. For transgenic lines, describe the transformation method, the number of independent lines analyzed and the generation upon which experiments were performed. For gene-edited lines, describe the editor used, the endogenous sequence targeted for editing, the targeting guide RNA sequence (if applicable) and how the editor was applied.*
Authentication	*Describe any authentication procedures for each seed stock used or novel genotype generated. Describe any experiments used to assess the effect of a mutation and, where applicable, how potential secondary effects (e.g. second site T-DNA insertions, mosiacism, off-target gene editing) were examined.*

ChIP-seq

Data deposition

☒ Confirm that both raw and final processed data have been deposited in a public database such as GEO.

☒ Confirm that you have deposited or provided access to graph files (e.g. BED files) for the called peaks.

Data access links
May remain private before publication.

The RNA-seq data can be found under the following link: https://www.ncbi.nlm.nih.gov/bioproject/PRJNA743896
The SSB-seq data can be found under the following link: https://www.ncbi.nlm.nih.gov/geo/query/acc.cgi?acc=GSE166963

Files in database submission

GSM5089933 Input_WT
GSM5089934 IP_WT_I
GSM5089935 IP_WT_II
GSM5089936 IP_WT_III
GSM5089937 Input_WT_Tal
GSM5089938 IP_WT_Tal_I
GSM5089939 IP_WT_Tal_II
GSM5089940 IP_WT_Tal_III
GSM5089941 Input_KO
GSM5089942 IP_KO_I
GSM5089943 IP_KO_II
GSM5089944 IP_KO_III
GSM5089945 Input_KO_Tal
GSM5089946 IP_KO_Tal_I
GSM5089947 IP_KO_Tal_II
SRS9404939 siControl_DMSO 3
SRS9404938 siControl_DMSO 2
SRS9404937 siControl_DMSO 1
SRS9404932 siControl_Tal 3
SRS9404933 siControl_Tal 2
SRS9404931 siControl_Tal 1

Genome browser session
(e.g. UCSC)

Provide a link to an anonymized genome browser session for "Initial submission" and "Revised version" documents only, to enable peer review. Write "no longer applicable" for "Final submission" documents.

Methodology

Replicates	n=3

Sequencing depth	Each sample in the SSB-seq experiments were sequenced to a depth of 40-70 mio. PE75 reads of which 30-50% fell into blacklistet regions, which led to an average of 26 mio uniquely mapped reads per sample.<br>toalRNA-seq samples were sequenced to a depth of at lease 90 mio reads using a single-end 100 bp approach
Antibodies	anti-DIG (Roche,11333089001)
Peak calling parameters	HOMER: findPeaks -style histone
Data quality	Depending on the condition, 15 - 35 k peaks were detected with a minimum fold enrichment over input >=4 and p-value over input <=1e-4 as well as a p-value over local region >=1e-4
Software	bowtie2 V2.3.4.2, picard tools V2.9.2, HOMER V4.11, STAR Aligner V2.6, HTseq V0.12.4<br>R V3.6 with packages and dependencies: Rsamtools V 2.10, bamsignals V1.22.0, edgeR V3.13, ChIPseeker V 1.26, DESeq2 version 1.26.0

Flow Cytometry

Plots

Confirm that:

- [ ] The axis labels state the marker and fluorochrome used (e.g. CD4-FITC).
- [ ] The axis scales are clearly visible. Include numbers along axes only for bottom left plot of group (a 'group' is an analysis of identical markers).
- [ ] All plots are contour plots with outliers or pseudocolor plots.
- [ ] A numerical value for number of cells or percentage (with statistics) is provided.

Methodology

Sample preparation	*Describe the sample preparation, detailing the biological source of the cells and any tissue processing steps used.*
Instrument	*Identify the instrument used for data collection, specifying make and model number.*
Software	*Describe the software used to collect and analyze the flow cytometry data. For custom code that has been deposited into a community repository, provide accession details.*
Cell population abundance	*Describe the abundance of the relevant cell populations within post-sort fractions, providing details on the purity of the samples and how it was determined.*
Gating strategy	*Describe the gating strategy used for all relevant experiments, specifying the preliminary FSC/SSC gates of the starting cell population, indicating where boundaries between "positive" and "negative" staining cell populations are defined.*

- [ ] Tick this box to confirm that a figure exemplifying the gating strategy is provided in the Supplementary Information.

Magnetic resonance imaging

Experimental design

Design type	*Indicate task or resting state; event-related or block design.*
Design specifications	*Specify the number of blocks, trials or experimental units per session and/or subject, and specify the length of each trial or block (if trials are blocked) and interval between trials.*
Behavioral performance measures	*State number and/or type of variables recorded (e.g. correct button press, response time) and what statistics were used to establish that the subjects were performing the task as expected (e.g. mean, range, and/or standard deviation across subjects).*

Acquisition

Imaging type(s)	*Specify: functional, structural, diffusion, perfusion.*
Field strength	*Specify in Tesla*
Sequence & imaging parameters	*Specify the pulse sequence type (gradient echo, spin echo, etc.), imaging type (EPI, spiral, etc.), field of view, matrix size, slice thickness, orientation and TE/TR/flip angle.*
Area of acquisition	*State whether a whole brain scan was used OR define the area of acquisition, describing how the region was determined.*

Diffusion MRI ☐ Used ☐ Not used

Preprocessing

Preprocessing software	*Provide detail on software version and revision number and on specific parameters (model/functions, brain extraction, segmentation, smoothing kernel size, etc.).*
Normalization	*If data were normalized/standardized, describe the approach(es): specify linear or non-linear and define image types used for transformation OR indicate that data were not normalized and explain rationale for lack of normalization.*
Normalization template	*Describe the template used for normalization/transformation, specifying subject space or group standardized space (e.g. original Talairach, MNI305, ICBM152) OR indicate that the data were not normalized.*
Noise and artifact removal	*Describe your procedure(s) for artifact and structured noise removal, specifying motion parameters, tissue signals and physiological signals (heart rate, respiration).*
Volume censoring	*Define your software and/or method and criteria for volume censoring, and state the extent of such censoring.*

Statistical modeling & inference

Model type and settings	*Specify type (mass univariate, multivariate, RSA, predictive, etc.) and describe essential details of the model at the first and second levels (e.g. fixed, random or mixed effects; drift or auto-correlation).*
Effect(s) tested	*Define precise effect in terms of the task or stimulus conditions instead of psychological concepts and indicate whether ANOVA or factorial designs were used.*

Specify type of analysis: ☐ Whole brain ☐ ROI-based ☐ Both

Statistic type for inference<br>(See Eklund et al. 2016)	*Specify voxel-wise or cluster-wise and report all relevant parameters for cluster-wise methods.*
Correction	*Describe the type of correction and how it is obtained for multiple comparisons (e.g. FWE, FDR, permutation or Monte Carlo).*

Models & analysis

n/a	Involved in the study
☐	☐ Functional and/or effective connectivity
☐	☐ Graph analysis
☐	☐ Multivariate modeling or predictive analysis

Functional and/or effective connectivity	*Report the measures of dependence used and the model details (e.g. Pearson correlation, partial correlation, mutual information).*
Graph analysis	*Report the dependent variable and connectivity measure, specifying weighted graph or binarized graph, subject- or group-level, and the global and/or node summaries used (e.g. clustering coefficient, efficiency, etc.).*
Multivariate modeling and predictive analysis	*Specify independent variables, features extraction and dimension reduction, model, training and evaluation metrics.*
